# Morphology of the Novel Basimandibular Gland in the Ant Genus *Strumigenys* (Hymenoptera, Formicidae)

**DOI:** 10.3390/insects12010050

**Published:** 2021-01-10

**Authors:** Chu Wang, Michael Steenhuyse-Vandevelde, Chung-Chi Lin, Johan Billen

**Affiliations:** 1 Zoological Institute, University of Leuven, Naamsestraat 59, Box 2466, B-3000 Leuven, Belgium; michael.steenhuyse@gmail.com (M.S.-V.); johan.billen@kuleuven.be (J.B.); 2 Department of Biology, National Changhua University of Education, Changhua 50007, Taiwan; cclin@cc.ncue.edu.tw

**Keywords:** exocrine glands, histology, ultrastructure, ants

## Abstract

**Simple Summary:**

Ants form a diverse group of social insects that are characterized by an overwhelming variety of exocrine glands, that play a key function in the communication system and social organization of the colony. Our focus goes to the genus *Strumigenys*, that comprise small slow-moving ants that mainly prey on springtails. We discovered a novel gland inside the mandibles of all 22 investigated species, using light and electron microscopy. As the gland occurs close to the base of the mandibles, we name it ‘basimandibular gland’ according to the putative description given to this mandible region in a publication by the eminent British ant taxonomist Barry Bolton in 1999. The gland exists in both workers and queens and appeared most developed in the queens of *Strumigenys mutica*. These queens in addition to the basimandibular gland also have a cluster of gland cells near the tip of their mandibles. The queens of this species enter colonies of other *Strumigenys* species and parasitize on them. We expect that the peculiar development of these glands inside the mandibles of these *S. mutica* queens plays a role in this parasitic lifestyle, and hope that future research can shed more light on the biology of these ants.

**Abstract:**

In 1999, Barry Bolton postulated the presence of a basimandibular gland in the mandibles in all species of the ant genus *Strumigenys*, solely based on scanning microscopy observations. We now confirm the presence of this putative gland in the proximal outer part of the mandibles of 22 investigated species by histological and ultrastructural examination, including 10 short- and 12 long-mandibulate species. All species have a basimandibular gland, that is formed by 15–25 µm thick epithelial cells and belongs to class-1 following the standard classification of insect exocrine glands. We consider it a novel gland because of its peculiar bowl-shape and special arrangement of the microvilli that are confined to large vacuolar spaces instead of reaching the cuticle. The gland is most pronounced in *S. mutica*, particularly in the queen. In addition to this gland, we also found scattered class-3 intramandibular gland cells in the mandibles. Queens of *S. mutica* are peculiar in having a cluster of these cells in the distal tip of their mandibles. As this species is a social parasite, further research is required to determine whether the development of these mandibular glands is related to its parasitic lifestyle.

## 1. Introduction

Social insects are well documented for their extraordinary variety of exocrine glands, with ants having the most developed exocrine system currently comprising a total of 89 glands [1]. The glands are positioned all over the body, including the antennae and legs [2,3]. Additionally, the mouthparts contain glandular tissue, with the mandibles especially housing several exocrine glands. The most common and universal is the mandibular gland that was first reported by Meinert back in 1860 [4]. The duct of this gland opens at the upper surface at the base of each mandible, although the main secretory part occurs inside the head capsule. Besides this ‘extramandibular’ gland, a variety of glands have also been described inside the mandibles. These belong either to the epithelial class-1 glands or the bicellular class-3 glands according to the standard classification by Noirot and Quennedey [5].

The myrmicine ant genus *Strumigenys* comprises around 830 species of small and slow-moving ants in both the tropics and subtropics [6,7]. All *Strumigenys* species are predators equipped with specialized prey-seizing mandibles and belong to two major groups: the short- and long-mandibulate species [8]. Although Ward [9] recently moved *Strumigenys* to the Attini, for a long time it represented the largest genus among the tribe Dacetonini [10]. A distinctive characteristic in this genus according to Bolton [10] is the presence of five exocrine glands, that represent exocrine structures exclusively restricted to *Strumigenys*. Of these five glands, only the apicofemoral and apicotibial glands have yet been histologically confirmed [3,11]. The basimandibular gland, the ventral scape gland and the mesopleural gland, however, remain undocumented. Using scanning microscopy, Bolton [10] observed a pale patch or streak structure at the ventral or lateroventral part near the base of the mandible. Without any histological studies, he concluded that this region corresponds with an exocrine structure and named it ‘basimandibular gland’. Through surveying several *Strumigenys* species for the presence of exocrine glands in the mandibles of workers and queens, we aim to verify the existence of this presumed basimandibular gland. Our study particularly focuses on *S. mutica*, as this species is a social parasite of other *Strumigenys* species such as *S. formosensis* or *S. solifontis*.

## 2. Materials and Methods

Workers and queens (and one male) belonging to 22 *Strumigenys* species were collected from their natural nests, representing both short- and long-mandibulate species (Table 1). The anterior parts of the heads were cut off under a Leica MZ 12.5 microscope (Leica, Wetzlar, Germany) and fixed in 2% cold glutaraldehyde in a 50 mM Na-cacodylate buffer at pH 7.3 for 12 h, followed by postfixation in 2% osmium tetroxide in the same buffer for one hour. After dehydration in a graded acetone series, the tissues were embedded in araldite. Serial 1 μm semithin sections were made using a diamond knife with a Leica EM UC6 ultramicrotome (Leica, Wetzlar, Germany). Sections for light microscopy were stained with methylene blue and thionin and observed under an Olympus BX-51 microscope (Olympus, Tokyo, Japan). Double-stained 70 nm thin sections were studied under a Zeiss EM900 electron microscope (Zeiss, Oberkochen, Germany). Samples for the scanning microscopy study were put on aluminium stubs with double-adhesive tape, coated with palladium and viewed under a JEOL JSM-6360 scanning microscope (JEOL Ltd., Tokyo, Japan).

## 3. Results

**Figure 1:**
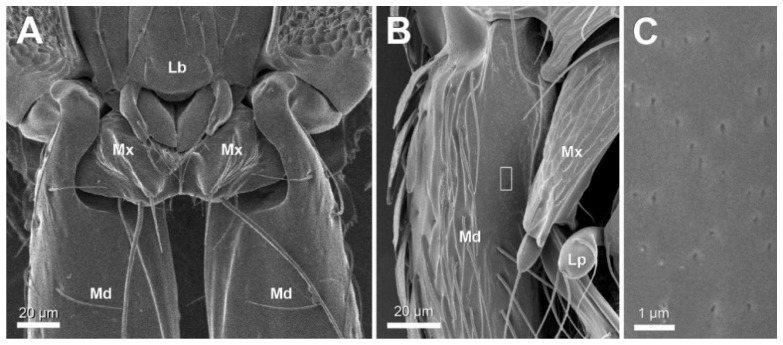
Ventral view scanning micrographs of mandible (Md). (**A**) *S. formosensis* worker. (**B**,**C**) *S. leptothrix* worker. Note smooth surface of proximal part of mandible, framed area in (**B**) appears enlarged in (**C**). Lb: labium, Lp: labial palp, Mx: maxilla.

Ventral view examination with scanning microscopy confirmed the existence of a smooth proximal portion of the ventral mandibular surface (Figure 1A,B). The cuticular surface in this area is devoid of hairs or any sculpturation as is found elsewhere on the mandible, and therefore appears uniformly smooth. Observation at higher magnification, however, reveals that the smooth area displays numerous scattered round to ovoid depressions of 50–120 nm with a density around 70 per 100 µm^2^ (Figure 1C).

**Figure 2:**
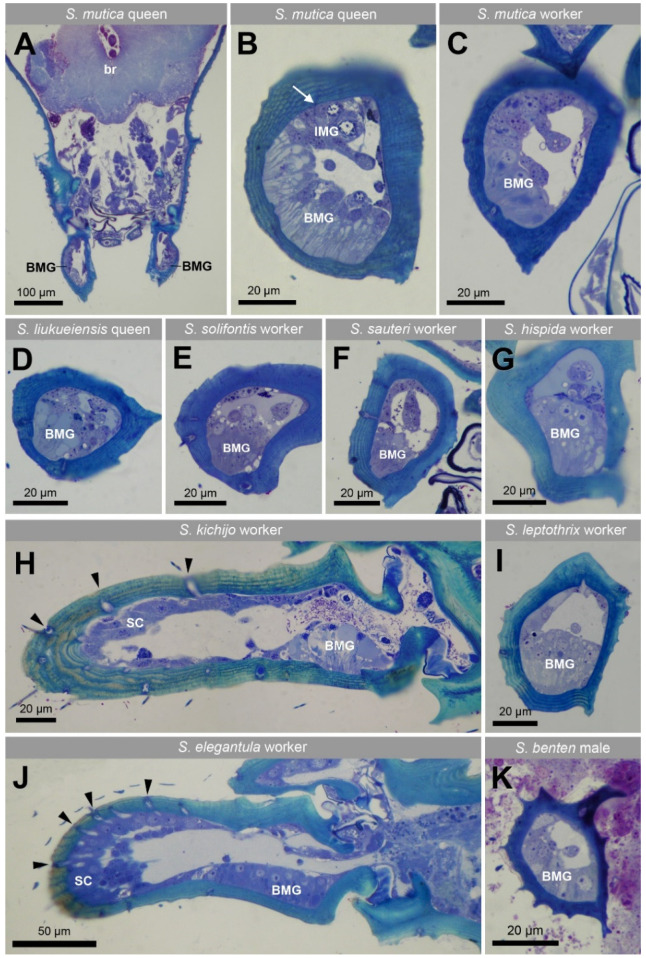
Semithin sections through head and mandibles of various *Strumigenys* species. (**A**) Frontal section through head of *S. mutica* queen, showing basimandibular gland (BMG) located at outer lateral side of mandibles, br: brain. (**B**,**C**) Cross sections through proximal part of left mandible of *S. mutica* queen and worker, showing both BMG and intramandibular gland (IMG). Arrow in B indicates duct of class-3 gland crossing cuticle. (**D**–**G**,**I**) Cross sections through the base of left mandible of several *Strumigenys* species. (**H**,**J**) Longitudinal sections through mandible of *S. kichijo* and *S. elegantula* workers, arrowheads indicate the large passage of sensillar cells through cuticle, SC: sensillar cells. (**K**) Cross section through mandible of *S. benten* male.

**Figure 3:**
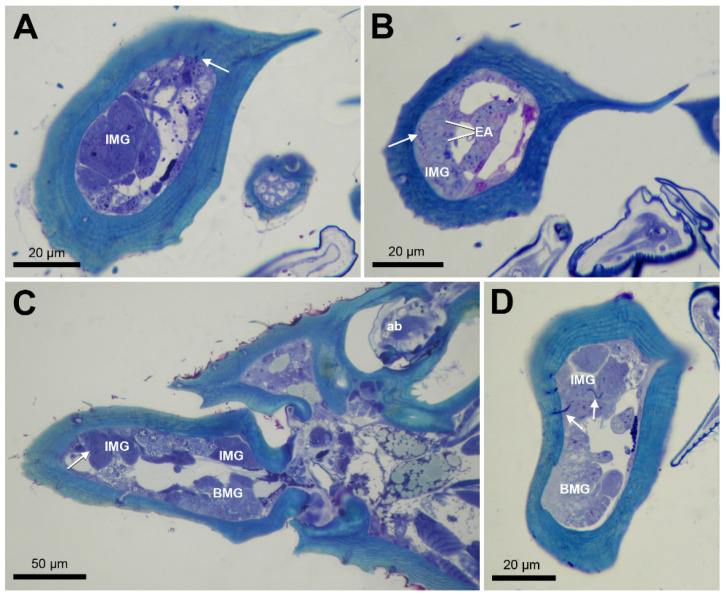
Semithin sections of the social parasite species *S. mutica*. (**A**,**B**) Cross section through mandible tip of queen (**A**) and worker (**B**), showing IMG cells, arrows indicate ducts opening through cuticle. EA: end apparatus. (**C**) Longitudinal section through head of queen, showing IMG dorsally and BMG ventrally, arrow indicates duct, ab: antenna base. (**D**) Cross section through proximal part of queen mandible, showing IMG dorsally and BMG ventrally, arrows indicate ducts.

Semithin sections through the mandibles of all 22 investigated species (Table 1) show the presence in both workers and queens of an epithelial gland which is located at the proximal ventrolateral side of each mandible (Figure 2 and Figure 3), which corresponds with the region of the smooth external cuticle. The cuticle overlaying the gland is approx. half as thick as the cuticle in the non-glandular region, which makes the gland look like a bowl-like extension into the cuticle (Figure 2 and Figure 3). Many large vesicles are apically found in the glandular cells. The epithelium is formed by cylindrical cells with a thickness of 15–25 µm. The extent of the glandular epithelium along the length of the mandible across species is similar for the species that belong to the short-mandibulate group (61.9 ± 12.3 µm, n = 14) and these belonging to the long-mandibulate group (62.8 ± 12.4, n = 12). In *S. mutica*, of which we had both castes available, the thickness of the epithelium is 23.11 ± 2.06 µm (n = 5) in queens, whereas in workers it measures 15.61 ± 2.41 µm (n = 8). In the single male (*S. benten*) that we had available, a similar glandular epithelium was also found under the ventral proximal part of the mandible (Figure 2K).

Apart from the class-1 basimandibular gland, we also observe scattered class-3 intramandibular gland cells with a diameter of around 10 µm in all species, of which the ducts may open through the upper or lower mandibular surface as small pores. This is also the case in *S. mutica*, in which the queens display the additional characteristic of having a compact cluster of approx. 10 such gland cells at the upper mandibular tip (Figure 3). The ducts of this gland transverse the cuticle to open dorsally near the inner margin of the mandibular tip (Figure 3A,C,D), whereas in workers, the ducts open more laterally at the outer side of the mandible (Figure 3B). The mandibles also contain several sensillar cells, that can be recognized by their large passage through the cuticle of 2–5 µm (Figure 2H,J).

**Figure 4:**
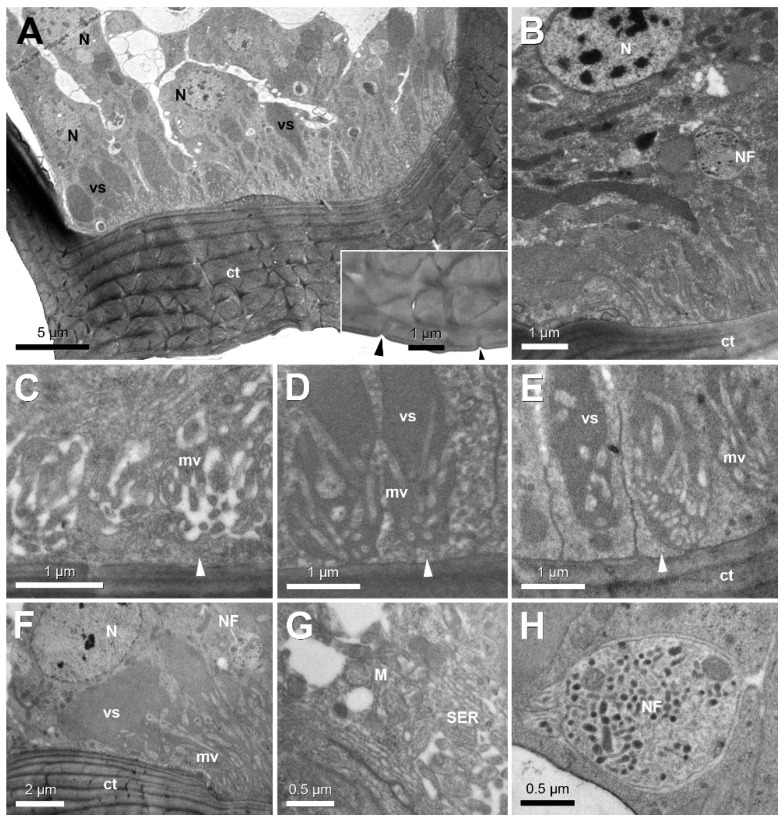
Electron micrographs of basimandibular gland. (**A**) General view of gland epithelium in *S. leptothrix* worker with basally located nuclei (N) and large vacuolar spaces (vs); inset shows detail of apical depressions on cuticular surface (arrowheads). (**B)** Gland epithelium in *S. lacunosa* worker. Note nerve fibre penetrating between gland cells (NF). (**C**–**E**) Details of apical cytoplasm and microvilli (mv) in workers of *S. minutula* (**C**), *S. elegantula* (**D**) and *S. leptothrix* (**E**), showing subcuticular cytoplasmic layer (arrowheads). (**F**) Gland epithelium in *S. mutica* worker showing large vacuolar spaces, microvilli and nerve fibre. (**G**) Central cytoplasm in *S. minutula* worker with mitochondria (M) and smooth endoplasmic reticulum (SER). (**H**) Detail of nerve fibre containing dark staining neurosecretory vesicles in *S. mutica* worker. ct: cuticle.

Ultrastructural examination of the glandular epithelium shows columnar cells with a basally located round nucleus with a diameter of 3–4 µm (Figure 4A,B). The horizontally layered cuticle overlaying the gland displays capricious transverse slits and spaces, that end up at the surface as minute pores around 100 nm (Figure 4A inset), and that correspond with the depressions observed with scanning microscopy (see Figure 1C). The apical portion of the epithelium contains numerous large spaces that are filled with secretion (Figure 4A,E,F). In their most apical part, these spaces contain microvilli. Most microvilli, however, do not reach the cuticle, but remain within the large space, which means the cuticle is lined underneath with a thin layer of apical cytoplasm (Figure 4C–E). The cytoplasm contains smooth endoplasmic reticulum and several mitochondria (Figure 4G). Wedged in between the glandular cells are one or two nerve fibres with a diameter of 1.5 µm that are loaded with small dark-staining neurosecretory vesicles of 50–80 nm (Figure 4H, see also Figure 4B,F). We did not find any muscle fibres associated with the basimandibular gland.

**Figure 5:**
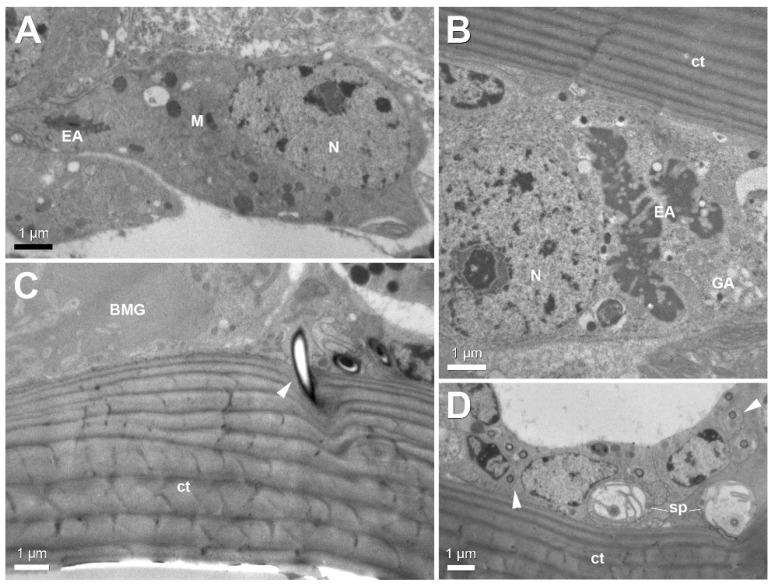
Electron micrographs of intramandibular gland. (**A**,**B)** General view of gland cell in worker of *S. mutica* (**A**) and *S. formosensis* (**B**), showing end apparatus (EA), mitochondria (M) and Golgi apparatus (GA). (**C)** Duct cell (arrowhead) penetrating ventral mandibular cuticle (ct) in *S. mutica* worker (BMG: basimandibular gland). (**D**) Narrow gland ducts (arrowheads) and broader sensillar processes (sp) adjacent to cuticle in *S. mutica* worker. ct: cuticle, N: nucleus.

The scattered intramandibular gland cells are characterized by their end apparatus, mitochondria and Golgi apparatus (Figure 5A,B). Their accompanying ducts cells open through the cuticle as narrow canals with a diameter of 0.25–0.5 µm (Figure 5C,D). The narrow gland ducts can be easily distinguished from the much broader sensillar passages through the cuticle (Figure 5D).

## 4. Discussion

The possibility to make histological sections through hard insect cuticle using epoxy resins as embedding medium instead of the traditional paraffin embedding has resulted in the discovery during the recent decades of many glandular structures inside the mandibles: the first gland described inside the mandibles of many ant species in different castes was simply termed ‘intramandibular gland’. It is formed by class-3 secretory cells underneath the outer margin of the mandibles that open mainly at the upper mandibular surface [12]. In *Oecophylla longinoda* [13] and *Aneuretus simoni* [14], this gland has ducts opening at both the upper and lower surface. A peculiar modification of the class-3 intramandibular gland is found in the rare agroecomyrmecine *Tatuidris tatusia*, in which all duct openings assemble and open through a sieve plate at the proximal ventral side near the inner edge of the mandibles [15]. In *Atta sexdens rubropilosa*, both class-1 and class-3 intramandibular glands have been reported [16]. In the pest ant *Brachyponera sennaarensis*, a large class-1 intramandibular gland appears underneath the distinctive mandibular pit that is a diagnostic character for this genus. The thick glandular epithelium extends along both the dorsal and ventral part of the mandibles [17]. Interestingly, an epithelial intramandibular gland has been described in *Strumigenys* (formerly *Pyramica*) *membranifera* [18], however, this gland is located at the inner distal tip of the mandibles which is very different from the position mentioned by Bolton [10]. In *Protanilla wallacei* workers and queens, the epithelial intramandibular gland with a proximal ventral position appears similar to the basimandibular gland we describe here [19], although it does not have the bowl-shape and also lacks the peculiar microvillar association with vacuolar spaces it has in *Strumigenys*. A conspicuous epithelial gland also occurs ventrally in the mandibles of *Proceratium japonicum* workers but is totally absent in the related *Discothyrea sauteri* (Billen, unpubl. obs.). This indicates the distribution of this gland among the Formicidae is unpredictable and phylogenetically unstable. Our findings in all 22 examined species of *Strumigenys*, however, do confirm the existence of the basimandibular gland as a novel exocrine structure and a diagnostic character for this genus [10]. Although ant males have much shorter mandibles than workers and queens, the presence of a similar glandular epithelium in the single male we had available illustrates the basimandibular gland represents a common feature in *Strumigenys* ants. Class-1 intramandibular glands are also found in stingless bees [20,21] and in neotropical Polistinae wasps [22].

The presence of smooth endoplasmic reticulum is indicative of the elaboration of a non-proteinaceous and hence possibly pheromonal function. Secretion reaches the mandibular surface after passing through tortuous intracuticular spaces, that connect to minute superficial slits. Such slits have also been described for other class-1 glands in ants such as the apicofemoral and apicotibial glands in *Strumigenys* [11] and several other epithelial glands in the legs of various species [3] as well as for the mandibular pit gland in *Brachyponera sennaarensis* [17]. A peculiar ultrastructural feature that has not been found so far in any of the many exocrine glands in social insects is the presence of microvilli that are associated with large vacuolar spaces without reaching the apical cuticle. The functional significance of this arrangement remains unknown. It is also striking to find one or two nerve fibres that are wedged in between the gland cells. Not much is known about the neural regulation mechanisms of secretion release in social insects, although the presence of the nerve fibres loaded with neurosecretory vesicles is an indication that neural activation is most probable. The appearance and size of these nerve fibres is in agreement with that of the axonal projections that have been found in the salivary glands of ticks [23]. In these glands, however, the axons provide the innervation of myoepithelial cells that upon contraction cause secretion release. The basimandibular gland, however, is not associated with any muscle fibres, which makes it more difficult to understand how release of secretion is achieved.

The function of the various intramandibular glands unfortunately remains undocumented. A study about the chemical composition of the intramandibular glands in *Neoponera villosa* suggests the compounds play a role of worker activity, and in the queen they may be related with a caste profile [24]. In *Oecophylla longinoda*, a peculiar behavior occurs when workers discover a new territory, a sugary food source, a prey or an alien ant, at such encounters they rub their mandibles onto the substrate as a marking signal to recruit their nestmates [13].

**Figure 6:**
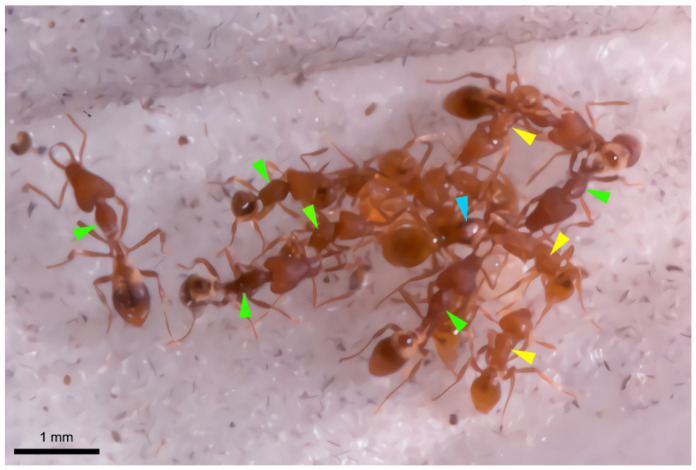
View of mixed lab colony with *S. mutica* queen (with enlarged thorax, blue arrowhead) surrounded by workers of *S. mutica* (yellow arrowheads) and *S. solifontis* (green arrowheads).

Special attention needs to be given to *Strumigenys mutica*, as this short-mandibulate species is an obligate social parasite in the nest of some long-mandibulate species such as *S. formosensis* or *S. solifontis* in Taiwan (C.-C. Lin, pers. comm.) or *S. lewisi* in Japan (F. Ito, pers. comm.). The young mated *S. mutica* queen enters a colony of one of these long-mandibulate species, where she eliminates the resident queen and takes over the reproductive command. Once the *S. mutica* queen has produced her own offspring, a mixed colony results with short-mandibulate *mutica* workers aside long-mandibulate workers of the host species (Figure 6). How the *mutica* queen manages to enter the host colony, how she kills the resident queen and how she makes herself accepted by the host workers, is totally unknown so far. Our finding of a prominent class-3 gland that opens at the tip of the mandibles of the *mutica* queen makes it tempting to speculate that this gland offers the parasite queen the weaponry to attack the resident queen, as the mandibles form an obvious tool for attacking an opponent. This hypothesis finds support in our finding of this gland only in the queens of *S. mutica*, whereas the eventual class-3 cells in *S. mutica* workers and in other *Strumigenys* species are less obvious and moreover occur in a more proximal position inside the mandibles. Another possible support is that also in the slave-making social parasite *Polyergus rufescens*, the usurper queen is equipped with obvious class-3 intramandibular gland cells near the inner tip of the sabre-shaped mandibles, although their function could not yet be elucidated [25]. Various glands and chemical strategies are used in this [25,26] as well as in other parasitic ants and this may also be the case in *Strumigenys*.

## 5. Conclusions

We confirm the putative existence of the basimandibular gland in the genus *Strumigenys* by histological and ultrastructural examination. The gland is considered to be a novel exocrine structure in ants. Although it appears somehow similar to the ‘intramandibular epithelial gland’ that has been described in *Protanilla wallacei* [19] and that may also occur in some other ant species, the novel basimandibular gland can be distinguished by its anatomical bowl-shape and peculiar arrangement of microvilli that are confined to large vesicular spaces instead of directly reaching the cuticle. In addition to the basimandibular gland, we suggest a potential function during colony foundation of a prominent distal class-3 intramandibular gland in the queens of the social parasite *S. mutica*.

## Figures and Tables

**Table 1:** Survey of ants studied with their collection locality and indication of number of workers (W), alate queens (AQ) and males (M) examined. All 22 species were studied histologically with serial semithin sections. Species indicated with ^S^ were also examined with SEM, species indicated with ^T^ were also examined with TEM.

	Species	Collection Locality	W	AQ	M
**short-mandibulate**	*S. benten* ^T^	Yuchi Township, Nantou County, Taiwan	3	0	1
	*S. canina*	Kobe, Japan	2	0	0
	*S. elegantula* ^T^	Yuchi Township, Nantou County, Taiwan	4	0	0
	*S. emmae* ^S^	Baihe District, Tainan City, Taiwan	2	0	0
	*S. hexamera*	Takamatsu, Japan	5	0	0
	*S. kichijo* ^T^	Lugu Township, Nantou County, Taiwan	4	0	0
	*S. leptothrix* ^S,T^	Ren’ai Township, Nantou County, Taiwan	4	0	0
	*S. membranifera*	Ren’ai Township, Nantou County, Taiwan	1	0	0
	*S. mutica* ^S,T^	Yuchi Township, Nantou County, Taiwan	8	5	0
	*S. sauteri*	Lugu Township, Nantou County, Taiwan	2	2	0
**long-mandibulate**	*S. chuchihensis*	Jianshi Township, Xinchu County, Taiwan	0	1	0
	*S. formosensis* ^S,T^	Ren’ai Township, Nantou County, Taiwan	6	0	0
	*S. hispida*	Yuchi Township, Nantou County, Taiwan	3	0	0
	*S. koningsbergeri*	Bogor Botanical Garden, Indonesia	1	0	0
	*S. lacunosa* ^T^	Lugu Township, Nantou County, Taiwan	2	1	0
	*S. liukueiensis*	Jiji Township, Nantou County, Taiwan	1	2	0
	*S. minutula* ^T^	Hengchun Township, Pingtung County, Taiwan	2	0	0
	*S. nanzanensis*	Lanyu Township, Taitung County, Taiwan	1	0	0
	*S. orchidensis*	Lanyu Township, Taitung County, Taiwan	1	0	0
	*S. perplexa*	Clyde Mountain, NSW, Australia	2	0	0
	*S. rogeri* ^S^	Jiji Township, Nantou County, Taiwan	0	2	0
	*S. solifontis*	Lugu Township, Nantou County, Taiwan	6	0	0

## Data Availability

All microscopy data are available in the Zoological Institute, LSSE Lab, University of Leuven, Belgium.
